# Equivalence testing of a newly developed interviewer-led telephone script for the EORTC QLQ-C30

**DOI:** 10.1007/s11136-021-02955-6

**Published:** 2021-07-20

**Authors:** Claire Piccinin, Madeline Pe, Dagmara Kuliś, James W. Shaw, Sally J. Wheelwright, Andrew Bottomley

**Affiliations:** 1 Quality of Life Department, EORTC, Brussels, Belgium; 2 Patient-Reported Outcomes Assessment, Bristol-Meyers Squibb, Lawrenceville, NJ USA; 3 Macmillan Survivorship Research Group, University of Southampton, Southampton, UK

**Keywords:** EORTC QLQ-C30, Health-related quality of life, Patient-reported outcomes, Core cancer symptoms, Equivalence testing, Voice script, Administration mode, Remote questionnaire administration

## Abstract

**Purpose:**

The European Organisation for Research and Treatment of Cancer (EORTC) Quality of Life-Core Questionnaire (QLQ-C30) is a widely used generic self-report measure of health-related quality of life (HRQOL) for cancer patients. However, no validated voice script for interviewer-led telephone administration was previously available. The aim of this study was to develop a voice script for interviewer administration via telephone.

**Methods:**

Following guidelines from the International Society for Pharmacoeconomics and Outcomes Research (ISPOR) PRO Mixed Modes Good Research Practices Task Force, a randomised cross-over equivalence study, including cognitive debriefing, was conducted to assess equivalence between paper and telephone administration modes. Assuming an expected intraclass correlation coefficient (ICC) of 0.70 and a minimally acceptable level of 0.50, a sample size of 63 was required.

**Results:**

Cognitive interviews with five cancer patients found the voice script to be clear and understandable. Due to a protocol deviation in the first wave of testing, only 26 patients were available for analyses. A second wave of recruitment was conducted, adding 37 patients (*n* = 63; mean age 55.48; 65.1% female). Total ICCs for mode comparison ranged from 0.72 (nausea and vomiting, 95% CI 0.48–0.86) to 0.90 (global health status/QoL, 95% CI 0.80–0.95; pain, 95% CI 0.79–0.95; constipation, 95% CI 0.80–0.95). For paper first administration, all ICCs were above 0.70, except nausea and vomiting (ICC 0.55; 95% CI 0.24–0.76) and financial difficulties (ICC 0.60; 95% CI 0.31–0.79). For phone first administration, all ICCs were above 0.70.

**Conclusions:**

The equivalence testing results support the voice script’s validity for administration of the QLQ-C30 via telephone.

**Supplementary Information:**

The online version contains supplementary material available at 10.1007/s11136-021-02955-6.

## Purpose

The European Organisation for Research and Treatment of Cancer (EORTC) Quality of Life-Core Questionnaire (QLQ-C30) [1] is currently one of the most widely used self-report measures of health-related quality of life (HRQOL) for cancer patients [2]. Patient reports using the EORTC QLQ-C30 are carried out using paper-and-pencil administration or through electronic methods. However, to increase the accessibility of the questionnaire across different research settings and populations (e.g. in patients that could be at risk of exclusion because of illiteracy), and to minimise the need for otherwise unnecessary clinic trips, the EORTC Quality of Life Group (QLG) set out to develop and validate a voice script for phone administration of the questionnaire. The voice script would incorporate all relevant elements of the QLQ-C30, along with additional instructions and text, where necessary, to facilitate its interviewer-led administration.

The EORTC QLQ-C30 was first released in 1987 by Aaronson et al. [3], and underwent further revisions leading to the development of its third version [1], which is still in use today. Comprised of 30 different items (questions), it is made up of eight multi-item functional (physical, role, emotional, cognitive, and social) and symptom (fatigue, pain, and nausea) scales, one global health status and quality of life (QOL) scale, and six single items (dyspnoea, insomnia, appetite loss, constipation, diarrhoea, and financial difficulties). Covering the majority of the core symptoms recommended for patient-reported outcome (PRO) measurement in cancer clinical trials [4], it is available for use in over 110 language versions, having undergone extensive testing to demonstrate its psychometric [5] and cultural [6] validity.

The majority of QLQ-C30 items (*n* = 28) are measured by a four-option Likert response scale that ranges from 1, indicating “not at all”, to 4, indicating “very much”, capturing the presence and/or severity of a symptom or issue and its impact on QOL. The final two items which make up the global health status and QOL scale are rated on a scale from 1 to 7, with 1 indicating “very poor” and 7 “excellent”. The time scale for all items is “during the past week” with the exception of the first 5 items (the physical functioning scale), for which no specific timeframe is used, given the intent to capture a more global impact on physical functioning, not limited to a 1-week recall period. All single items and multi-item scales in the questionnaire are scored and transformed onto a 0–100 scale, with higher scores for the functional and global health status/QOL scales indicating higher levels of functioning and QOL, and higher scores for symptom scales and single items indicating a higher degree of symptomatology and problems.

In addition to its frequent use in cancer research and clinical trials [2, 7], the QLQ-C30 is being increasingly used for monitoring purposes in clinical practice [8]. By providing a direct means of measuring core symptoms and issues from the patient’s perspective, the QLQ-C30 provides clinically meaningful information, distinct from that offered by clinical markers and clinicians’ ratings [2, 7, 9]. In 2018, the EORTC QLG published guidelines to help facilitate the use and migration of EORTC questionnaires into electronic PRO (ePRO) formats (e.g. computer, tablet) [10]. A computerised adaptive testing (CAT) version of the QLQ-C30, the EORTC CAT Core [11], is also available, and consists of dynamic item banks which correspond with the QLQ-C30’s 14 functional and symptom domains.

The purpose of this study was to pilot test the provisional QLQ-C30 phone script through cognitive debriefing interviews to ensure its acceptability and relevance, amending it if needed, and to subsequently validate the QLQ-C30 phone-administered version by carrying out equivalence testing between the paper and phone administration modes in a population of patients actively undergoing cancer treatment. An intraclass correlation coefficient (ICC) of ≥ 0.70, the recommended threshold to demonstrate equivalence between various modes of administration, was employed in this study for the purpose of equivalence testing [12]. Previous research supports the use of ICC ≥ 0.70, as demonstrated in studies by Lundy et al. [13, 14], in which an interactive voice response (IVR) version of the QLQ-C30 was developed. Similarly, in an equivalence study aimed at comparing tablet computer, IVR, and paper-based administration of the PRO-CTCAE [15], the degree of mode equivalence was assessed using ICC ≥ 0.70.

Although previous work conducted by Lundy et al. demonstrated the equivalence of an IVR version of the QLQ-C30 to its paper administration [13, 14], this is the first project aimed at validating a voice script for phone administration of the QLQ-C30 by an interviewer. A considerable body of research comparing paper to screen-based (e.g. tablet, computer) administration of PROs has demonstrated high levels of reliability between both modes [16–18] but less work has compared paper administration to auditory modes (e.g. IVR, phone interview). Still, the existing research suggests that equivalence can be established between paper and phone PRO administration [13, 15].

## Methods

Patient recruitment and data collection, management, and analysis were subcontracted to contract research organisation (CRO) Mapi/ICON plc who provided a final report to the EORTC detailing the methodology and findings. Study approval was obtained in the United Kingdom (UK) by the Quorum Review independent review board.

### Sample

Recruitment was carried out through a UK-based recruitment agency (Global Perspectives Limited) and patients were eligible to participate if they were 18 years or older, currently receiving cancer treatment as confirmed by a clinician, able to read and understand English, voluntarily agreed to participate in the study, and provided written informed consent.

#### Pilot-testing

Five patients were interviewed to test the acceptability, understanding, and relevance of the instructions for the QLQ-C30 voice script. Given that the QLQ-C30 has already been extensively tested and validated, and pilot-testing of the script had the aim of ensuring that the inclusion of additional text (e.g. instructions) was understandable, 5 patients was determined to be an acceptable number.

#### Equivalence testing

In addition to the previously described eligibility criteria, patients in the equivalency testing were required to have no changes in treatment planned between the paper and phone version completion. To support equivalence between paper-and-pen and phone administration modes using an ICC ≥ 0.70 and a minimally acceptable level of 0.50, a sample size of 63 patients was required [12]. Two waves of recruitment were conducted. In the first wave, 50 patients were recruited, the appropriate number for an equivalence threshold of ICC ≥ 0.90. Since protocol deviations were observed in which only 26 patients completed the paper and phone versions of the QLQ-C30 within the pre-specified 2-day timeframe, a second wave of recruitment was therefore conducted to address these limitations. The deviation relative to the 2-day time frame was due to issues with mailing of the questionnaires, and missing time stamps. The initial decision to use an equivalence threshold of ICC ≥ 0.90 garnered significant discussion among the research team members and CRO. Following the protocol deviation, it was deemed an appropriate moment to adapt the criteria to the widely used ICC ≥ 0.70. As such, thirty-seven additional patients were recruited based on the same eligibility criteria, bringing the total sample size to 63.

### Study design

#### Pilot-testing

Patients’ interviews were conducted by trained qualitative researchers and audio-recorded for the purpose of analysis. Interviews lasted approximately 60 min and were based on a study-specific interview guide, which contained a summary of the methods to conduct the interview, along with semi-structured questions. The guide also contained questions regarding demographic and clinical variables to capture during the interview. Patients’ responses were recorded anonymously on a grid. The QLQ-C30 phone script was subsequently revised accordingly, and the updated version was used for equivalence testing. The interview recordings were destroyed after completion of the analysis, with an anonymised copy of the recordings retained for the study files.

#### Equivalence testing

A randomised, cross-over design was used to compare the self-administered paper version and the phone-administered version of the QLQ-C30 in patients currently receiving treatment for cancer, following recommendations as set out in the International Society for Pharmacoeconomics and Outcomes Research (ISPOR) PRO Mixed Methods Task Force [19]. Patients were randomised (1:1) to complete either the paper or the phone-administered version first. Randomisation was conducted using a random number generator. After providing informed consent, each patient completed a brief socio-demographic and clinical form. Depending on randomisation, patients were then asked to either complete the paper version of the QLQ-C30 and return it to the recruitment agency in a prepaid envelope or respond by phone to the questionnaire following the phone script as presented by the interviewer, a trained qualitative researcher. The interviewers recorded patients’ responses on a paper version of the QLQ-C30. The paper version of the QLQ-C30 was estimated to take approximately 30 min to complete and administration time for the phone version was recorded for each patient. Any comments or observations made by the patient during the phone administration were recorded on a feedback form.

Two days after the first completion of the QLQ-C30, patients were asked to complete it again using the other mode of administration. The date of completion of the paper version was noted for each patient, to assess compliance with the pre-specified 2-day time frame. For patients who completed the phone interview first, the recruitment agency waited for confirmation of interview completion from the study team before sending the paper version by post.

### Data analysis

Patients were described in terms of clinical and socio-demographic variables, as reported during the phone interview (pilot-testing) or on the socio-demographic/clinical form (equivalence testing). Age, gender, educational status, and disease history were reported. All data processing and analyses were performed with SAS® software for Windows, Version 9.2 or later (SAS Institute, Inc., Cary, NC, USA).

#### Pilot-testing

Feedback from patients was compiled in an analysis grid, and reported per patient based on a qualitative assessment of the questionnaire, its instructions and individual items, with any additional comments also recorded.

#### Equivalence testing

All patients who met the inclusion criteria and completed enough items in the QLQ-C30 questionnaire during each administration for each domain to be scored were included in the equivalence testing analysis. Responses to items from the QLQ-C30 were described based on completion and distribution of responses per administration mode. Missing data were described in terms of number and percent of missing responses per item along with number and percent of missing items per patient, including the number of patients with at least one missing item. Continuous variables were described based on their frequency, mean, standard deviation, median, first and third quartiles, and minimum and maximum values. Categorical variables were described based on the frequency and percentage of each response choice, with missing data included in the calculation of percentage.

Equivalence testing was performed at both the item and domain score levels, with the primary objective to evaluate equivalence at the score level between both modes of administration using ICC [20]. A two-way mixed effects, consistency, single rater/measurement approach, described by Fleiss et al. [20], was used to calculate ICCs and their confidence intervals. The widely used benchmark of ICC of ≥ 0.70 was used [21], with ICC values between 0.75 and 0.90 indicating good agreement and values greater than 0.90 indicating excellent agreement [22]. Weighted kappa coefficients [23] were used to assess the extent to which both administration modes produced the same responses by patients to the QLQ-C30 items (results are reported in Online Appendix A). Following Fleiss’ guidelines [24], a kappa value greater than 0.75 was characterised as excellent, 0.40–0.75 as fair to good, and less than 0.40 as poor. Mean differences in item-level scores were also calculated and are displayed in Online Appendix B.

To ensure robustness of results between the two waves of recruitment, a sensitivity analysis was conducted to compare the ICC values between patients included prior to the study amendment (first wave of recruitment: *n* = 26) and those included after (second wave of recruitment: *n* = 37) using scores from the paper and phone administration modes of the QLQ-C30. Additional sensitivity analyses were conducted on the full group of patients included in the equivalence testing (*n* = 63) to compare ICC scores by age (< 60 vs. ≥ 60) and gender.

## Results

### Sample

#### Pilot-testing

Five patients (three males and two females) with a mean age of 51 years completed the pilot-testing interviews. Patients had either liver, testicular, bowel cancer, or lymphoma, and one patient had breast, lung, and bowel cancer, as well as secondary liver cancer. More details regarding demographic and clinical characteristics are provided in Table 1.

**Table 1:** Demographic and clinical characteristics of the pilot-testing population (*n* = 5)

Variable	Pilot-testing sample (*n* = 5)
Age, years	
Median	51
Min–max	(29–64)
Gender, *n*	
Male/female	3/2
Living status, *n*	
Living as a couple	3
Living alone	2
Occupation, *n*	
Full- or part-time employment	4
Retired	1
Education, *n*	
Left high school with no qualifications	1
Completed high school with qualifications	3
Other	2
Type of cancer, *n*	
Breast*	1
Lung*	1
Bowel*	2
Other*	4
Therapy	
Surgery**	1
Chemotherapy**	3
Other	2

*One patient had breast, lung and bowel cancer, and secondary liver cancer

**One patient had both surgery and chemotherapy

#### Equivalence testing

Sixty-three patients (26 from the first wave and 37 from the second wave) made up the total sample included in the equivalence testing. Patients had a mean age of 55 years and 65% were female. Almost half of the sample (48%) was employed full- or part-time and 76% of patients were living as a couple. Education levels varied with 41% of patients having obtained a bachelor’s or postgraduate degree. Breast cancer was the most common disease type, reported in 29% of patients, followed by prostate (11%), lung (10%), and bowel (6%) cancers. A large proportion of patients (41%) reported “other” disease types. The majority of patients were undergoing chemotherapy (25%) or hormone therapy (16%) and other types of treatment included surgery (11%), radiotherapy (10%), biological therapy (13%), mixed therapy (8%) and “other” types of treatment (18%). Detailed demographic and clinical characteristics are provided in Table 2, presented to indicate patients who completed the paper (*n* = 31) or phone (*n* = 32) versions of the QLQ-C30 first.

**Table 2:** Demographic and clinical characteristics of the equivalence testing population (*n* = 63)

Variable	Randomisation group		Total (*n* = 63)
	Paper version first (*n* = 31)	Phone version first (*n* = 32)	
Age, years			
* n* (Missing)	31 (0)	32 (0)	63 (0)
Mean (SD)	56.84 (13.83)	54.16 (11.36)	55.48 (12.61)
Median	59.00	54.50	58.00
Min–max	24.00–78.00	31.00–79.00	24.00–79.00
Gender, *n* (%)			
Male	12 (38.7%)	10 (31.3%)	22 (34.9%)
Female	19 (61.3%)	22 (68.8%)	41 (65.1%)
Living status, *n* (%)			
Living alone	6 (19.4%)	3 (9.4%)	9 (14.3%)
Living as a couple	23 (74.2%)	25 (78.1%)	48 (76.2%)
Other	2 (6.5%)	4 (12.5%)	6 (9.5%)
Occupation, *n* (%)			
Full/part-time employment	11 (35.5%)	19 (59.4%)	30 (47.6%)
Homemaker/housewife	1 (3.2%)	2 (6.3%)	3 (4.8%)
Student	1 (3.2%)	0 (0.0%)	1 (1.6%)
Unemployed	2 (6.5%)	1 (3.1%)	3 (4.8%)
Retired	9 (29.0%)	6 (18.8%)	15 (23.8%)
Other	4 (12.9%)	4 (12.5%)	8 (12.7%)
Full/part-time employment and Homemaker	3 (9.7%)	0 (0.0%)	3 (4.8%)
Education level, *n* (%)			
Left high school with no qualifications	2 (6.5%)	4 (12.5%)	6 (9.5%)
Completed high school with qualifications	10 (32.3%)	12 (37.5%)	22 (34.9%)
Bachelor’s degree	6 (19.4%)	4 (12.5%)	10 (15.9%)
Post-graduate degree	8 (25.8%)	8 (25.0%)	16 (25.4%)
Other	5 (16.1%)	4 (12.5%)	9 (14.3%)
Type of cancer, *n* (%)			
Breast	10 (32.3%)	8 (25.0%)	18 (28.6%)
Prostate	5 (16.1%)	2 (6.3%)	7 (11.1%)
Lung	5 (16.1%)	1 (3.1%)	6 (9.5%)
Bowel	1 (3.2%)	3 (9.4%)	4 (6.3%)
Other	9 (29.0%)	17 (53.1%)	26 (41.3%)
Kidney (metastatic)	0 (0.0%)	1 (3.1%)	1 (1.6%)
Lung (non-small cell)	1 (3.2%)	0 (0.0%)	1 (1.6%)
Treatment*, *n* (%)			
Surgery	4 (12.9%)	3 (9.4%)	7 (11.1%)
Chemotherapy	9 (29.0%)	7 (21.9%)	16 (25.4%)
Radiotherapy	2 (6.5%)	4 (12.5%)	6 (9.5%)
Hormone therapy	6 (19.4%)	4 (12.5%)	10 (15.9%)
Biological therapy	2 (6.5%)	6 (18.8%)	8 (12.7%)
Other	6 (19.4%)	5 (15.6%)	11 (17.5%)
Mixed	2 (6.5%)	3 (9.4%)	5 (7.9%)

*Patients could select more than one treatment

Participants in both waves of testing were largely similar, with more considerable differences observed based on treatment type. Whereas 16.2% of patients reported undergoing surgery in the second wave of testing, only 3.8% reported it in the first wave. Moreover, no patients reported use of biological therapy in the second wave of testing, while 30.8% of patients reported it in the first wave. The full comparison of socio-demographic differences is presented in Table 3.

**Table 3:** Socio-demographic differences between patients from the two study waves

Variable	Patients		Total (*n* = 63)
	Second wave of patients (*n* = 37)	First wave of patients (*n* = 26)	
Age, years			
* n* (Missing)	37 (0)	26 (0)	63 (0)
Mean (SD)	52.51 (12.79)	59.69 (11.27)	55.48 (12.61)
Median	56.00	59.50	58.00
Min–max	24.00–76.00	36.00–79.00	24.00–79.00
Gender, *n* (%)			
Male	8 (21.6%)	14 (53.8%)	22 (34.9%)
Female	29 (78.4%)	12 (46.2%)	41 (65.1%)
Living status, *n* (%)			
Living alone	7 (18.9%)	2 (7.7%)	9 (14.3%)
Living as a couple	25 (67.6%)	23 (88.5%)	48 (76.2%)
Other	5 (13.5%)	1 (3.8%)	6 (9.5%)
Occupation, *n* (%)			
Full/part-time employment	17 (45.9%)	13 (50.0%)	30 (47.6%)
Homemaker/housewife	3 (8.1%)	0 (0.0%)	3 (4.8%)
Student	1 (2.7%)	0 (0.0%)	1 (1.6%)
Unemployed	3 (8.1%)	0 (0.0%)	3 (4.8%)
Retired	7 (18.9%)	8 (30.8%)	15 (23.8%)
Other	3 (8.1%)	5 (19.2%)	8 (12.7%)
Full/part-time employment and homemaker	3 (8.1%)	0 (0.0%)	3 (4.8%)
Education level, *n* (%)			
Left high school with no qualifications	3 (8.1%)	3 (11.5%)	6 (9.5%)
Completed high school with qualifications	12 (32.4%)	10 (38.5%)	22 (34.9%)
Bachelor’s degree	7 (18.9%)	3 (11.5%)	10 (15.9%)
Post-graduate degree	9 (24.3%)	7 (26.9%)	16 (25.4%)
Other	6 (16.2%)	3 (11.5%)	9 (14.3%)
Type of cancer, *n* (%)			
Breast	11 (29.7%)	7 (26.9%)	18 (28.6%)
Prostate	2 (5.4%)	5 (19.2%)	7 (11.1%)
Lung	3 (8.1%)	3 (11.5%)	6 (9.5%)
Bowel	2 (5.4%)	2 (7.7%)	4 (6.3%)
Other	17 (45.9%)	9 (34.6%)	26 (41.3%)
Kidney (metastatic)	1 (2.7%)	0 (0.0%)	1 (1.6%)
Lung (non-small cell)	1 (2.7%)	0 (0.0%)	1 (1.6%)
Treatment*			
Surgery	6 (16.2%)	1 (3.8%)	7 (11.1%)
Chemotherapy	11 (29.7%)	5 (19.2%)	16 (25.4%)
Radiotherapy	2 (5.4%)	4 (15.4%)	6 (9.5%)
Hormone therapy	4 (10.8%)	6 (23.1%)	10 (15.9%)
Biological therapy	0 (0.0%)	8 (30.8%)	8 (12.7%)
Other	11 (29.7%)	0 (0.0%)	11 (17.5%)
Mixed	3 (8.1%)	2 (7.7%)	5 (7.9%)

*Patients could select more than one treatment

### Pilot-testing

All patients considered the instructions in the phone script to be clear and straightforward. Three comments were raised concerning the time and response scales of the questionnaire. Two patients made comments regarding the time scales, specifying that “during the past week” was too short of a time frame. However, these comments deviated from the source questionnaire and were thus not integrated into the script. One patient suggested numbering the response options from 1 to 4, for clarity. After discussion with the study team, numbers 1 to 4 were added to the response options in the phone script, thereby creating the final version of the phone script in UK English.

### Equivalence testing

All patients from both testing waves (*n* = 63) completed all items in both the paper and phone versions of the QLQ-C30 and there were no missing data. Table 4 displays the results of the equivalence testing for QLQ-C30 scores between the paper and phone administration modes. Total ICC values were above the 0.70 threshold and ranged from 0.72 for nausea and vomiting (95% CI 0.48–0.86) to 0.90 for global health status/QoL (95% CI 0.80–0.95), cognitive functioning (95% CI 0.80–0.95), and pain (95% CI 0.79–0.95). ICCs were also calculated to compare paper first versus phone first administration. For paper first administration, the ICCs ranged from 0.55 for nausea and vomiting (95% CI 0.24–0.76) to 0.90 for appetite loss (95% CI 0.80–0.95) and constipation (95% CI 0.81–0.95), with only two scores, nausea and vomiting and financial difficulties (ICC 0.60; 95% CI 0.31−0.79), falling below the 0.70 threshold. For phone first administration, all ICC values were above the 0.70 threshold and ranged from 0.79 for dyspnoea (95% CI 0.60–0.89) to 0.94 for physical functioning (95% CI 0.87–0.97). Results for equivalence testing at the single item level are displayed in Online Appendix A.

**Table 4:** Equivalence testing for QLQ-C30 scores

QLQ-C30 scores	All patients		Total (n = 63)
	Paper first (*n* = 31)	Phone first (*n* = 32)	
Global health status score			
ICC	0.89	0.91	0.90
95% CI	0.79–0.95	0.81–0.95	0.80–0.95
Physical functioning score			
ICC	0.81	0.94	0.89
95% CI	0.64–0.90	0.89–0.97	0.79–0.95
Role functioning score			
ICC	0.76	0.89	0.84
95% CI	0.56–0.88	0.78–0.94	0.68–0.92
Emotional functioning score			
ICC	0.79	0.92	0.86
95% CI	0.60–0.89	0.84–0.96	0.73–0.93
Cognitive functioning score			
ICC	0.85	0.94	0.90
95% CI	0.71–0.92	0.87–0.97	0.80–0.95
Social functioning score			
ICC	0.76	0.85	0.79
95% CI	0.55–0.87	0.70–0.92	0.59–0.89
Fatigue score			
ICC	0.76	0.82	0.79
95% CI	0.56–0.88	0.66–0.91	0.59–0.89
Nausea and vomiting			
ICC	0.55	0.91	0.72
95% CI	0.24–0.76	0.83–0.96	0.48–0.86
Pain			
ICC	0.89	0.90	0.90
95% CI	0.78–0.94	0.80–0.95	0.79–0.95
Dyspnoea			
ICC	0.78	0.79	0.76
95% CI	0.59–0.89	0.60–0.89	0.55–0.88
Insomnia			
ICC	0.87	0.83	0.84
95% CI	0.74–0.93	0.68–0.92	0.69–0.92
Appetite loss			
ICC	0.90	0.82	0.85
95% CI	0.80–0.95	0.65–0.91	0.71–0.93
Constipation			
ICC	0.90	0.90	0.90
95% CI	0.81–0.95	0.80–0.95	0.80–0.95
Diarrhoea			
ICC	0.77	0.85	0.80
95% CI	0.58–0.88	0.71–0.92	0.62–0.90
Financial difficulties			
ICC	0.60	0.87	0.73
95% CI	0.31–0.79	0.75–0.93	0.49–0.86

Mean differences in domain-level scores were assessed between administration modes and are shown in Table 5. Results for mean differences at the item level are displayed in Online Appendix B. At the domain level, differences between modes were minimal in absolute magnitude, ranging from 0.00 to 11.00 points.

**Table 5:** Mean differences in multi-item scores between paper-and-pen versions

QLQ-C30 scores	All patients	
	Paper first (*n* = 31)	Phone first (*n* = 32)
Global health status		
Mean (SD)	− 0.67 (9.01)	− 0.52 (8.71)
Min–max	− 16.67 to 33.33	− 25.00 to 25.00
Physical functioning		
Mean (SD)	− 2.37 (8.35)	1.04 (6.36)
Min–max	− 20.00 to 20.00	− 6.67 to 26.67
Role functioning		
Mean (SD)	− 0.54 (15.80)	5.73 (14.42)
Min–max	− 33.33 to 33.33	− 16.67 to 33.33
Emotional functioning		
Mean (SD)	0.54 (14.26)	4.43 (10.37)
Min–max	− 25.00 to 33.33	− 25.00 to 25.00
Cognitive functioning		
Mean (SD)	1.43 (10.92)	4.17 (7.98)
Min–max	− 16.67 to 33.33	− 16.67 to 22.22
Social functioning		
Mean (SD)	− 9.68 (19.61)	1.04 (15.23)
Min–max	− 50.00 to 33.33	− 33.33 to 33.33
Fatigue		
Mean (SD)	0.72 (16.71)	− 6.25 (14.09)
Min–max	− 44.44 to 33.33	− 44.44 to 22.22
Nausea and vomiting		
Mean (SD)	4.30 (12.89)	− 2.08 (5.60)
Min–max	0.00 to 66.67	− 16.67 to 0.00
Pain		
Mean (SD)	1.08 (14.23)	1.56 (13.63)
Min–max	− 16.67 to 50.00	− 16.67 to 33.33
Dyspnoea		
Mean (SD)	2.15 (17.07)	− 8.33 (14.66)
Min–max	− 33.33 to 66.67	− 33.33 to 0.00
Insomnia		
Mean (SD)	1.08 (16.06)	− 7.29 (20.27)
Min–max	− 33.33 to 33.33	− 66.67 to 33.33
Appetite loss		
Mean (SD)	3.23 (10.02)	− 0.00 (14.66)
Min–max	0.00 to 33.33	− 33.33 to 33.33
Constipation		
Mean (SD)	− 1.08 (10.48)	− 2.08 (14.51)
Min–max	− 33.33 to 33.33	− 33.33 to 33.33
Diarrhoea		
Mean (SD)	− 1.08 (20.15)	− 4.17 (14.04)
Min–max	− 100.00 to 33.33	− 33.33 to 33.33
Financial difficulties		
Mean (SD)	10.75 (30.29)	5.21 (12.30)
Min–max	− 66.67 to 66.67	0.00 to 33.33

The mean time for completion of the phone version of the QLQ-C30 was 8.6 ± 1.9 min and 39 participants (62%) made comments or asked questions during the interview.

Sensitivity analyses comparing patients included before the study amendment (*n* = 26) with those included after (*n* = 37) revealed significant differences (i.e. 95% CI overlapping) only for the nausea and vomiting ICC, which was lower in the first wave of patients, and the constipation ICC, which was lower in the second wave. The full results are displayed in Table 6.

**Table 6:** Sensitivity analysis of ICC scores by testing waves

QLQ-C30 scores	All patients		Total (*n* = 63)
	1st wave (*n* = 26)	2nd wave (*n* = 37)	
Global health status score			
ICC	0.92	0.89	0.90
95% CI	0.83–0.96	0.79–0.95	0.80–0.95
Physical functioning score			
ICC	0.86	0.93	0.89
95% CI	0.72–0.93	0.58–0.96	0.79–0.95
Role functioning score			
ICC	0.76	0.77	0.84
95% CI	0.55–0.88	0.57–0.88	0.68–0.92
Emotional functioning score			
ICC	0.84	0.87	0.86
95% CI	0.69–0.92	0.74–0.94	0.73–0.93
Cognitive functioning score			
ICC	0.90	0.90	0.90
95% CI	0.81–0.95	0.79–0.95	0.80–0.95
Social functioning score			
ICC	0.82	0.75	0.79
95% CI	0.67–0.91	0.53–0.87	0.59–0.89
Fatigue score			
ICC	0.77	0.82	0.79
95% CI	0.57–0.88	0.64–0.91	0.59–0.89
Nausea and vomiting			
ICC	0.47	0.96	0.72
95% CI	0.14–0.71	0.90–0.98	0.48–0.86
Pain			
ICC	0.85	0.94	0.90
95% CI	0.71–0.93	0.87–0.97	0.79–0.95
Dyspnoea			
ICC	0.73	0.82	0.76
95% CI	0.50–0.86	0.65–0.91	0.55–0.88
Insomnia			
ICC	0.78	0.89	0.84
95% CI	0.59–0.89	0.80–0.95	0.69–0.92
Appetite loss			
ICC	0.85	0.85	0.85
95% CI	0.70–0.92	0.71–0.93	0.71–0.93
Constipation			
ICC	0.98	0.84	0.90
95% CI	0.95–0.99	0.70–0.92	0.80–0.95
Diarrhoea			
ICC	0.72	0.90	0.80
95% CI	0.49–0.86	0.79–0.95	0.62–0.90
Financial difficulties			
ICC	0.52	0.79	0.73
95% CI	0.20–0.74	0.60–0.90	0.49–0.86

The results of additional sensitivity analyses to assess possible differences in scores based on age (< 60 versus ≥ 60) and gender are displayed in Tables 7 and 8.

**Table 7:** Sensitivity analysis of ICC scores by age above and below 60

QLQ-C30 scores	All patients	
	Age < 60 (*n* = 40)	Age ≥ 60 (*n* = 23)
Global health status score		
ICC	0.89	0.90
95% CI	0.78–0.94	0.81–0.95
Physical functioning score		
ICC	0.95	0.77
95% CI	0.89–0.97	0.57–0.88
Role functioning score		
ICC	0.84	0.81
95% CI	0.69–0.92	0.64–0.90
Emotional functioning score		
ICC	0.88	0.81
95% CI	0.76–0.94	0.65–0.90
Cognitive functioning score		
ICC	0.90	0.88
95% CI	0.80–0.95	0.77–0.94
Social functioning score		
ICC	0.81	0.69
95% CI	0.65–0.91	0.45–0.84
Fatigue score		
ICC	0.80	0.75
95% CI	0.61–0.90	0.55–0.87
Nausea and vomiting		
ICC	0.63	0.92
95% CI	0.35–0.81	0.84–0.96
Pain		
ICC	0.89	0.89
95% CI	0.79–0.95	0.80–0.95
Dyspnoea		
ICC	0.79	0.72
95% CI	0.61–0.90	0.49–0.85
Insomnia		
ICC	0.88	0.72
95% CI	0.76–0.94	0.49–0.85
Appetite loss		
ICC	0.92	0.71
95% CI	0.85–0.96	0.48–0.85
Constipation		
ICC	0.87	0.94
95% CI	0.75–0.94	0.88–0.97
Diarrhoea		
ICC	0.68	0.95
95% CI	0.42–0.83	0.90–0.97
Financial difficulties		
ICC	0.75	0.54
95% CI	0.54–0.87	0.23–0.75

**Table 8:** Sensitivity analysis of ICC scores by gender

QLQ-C30 scores	All patients	
	Male (*n* = 22)	Female (*n* = 41)
Global health status score		
ICC	0.92	0.87
95% CI	0.84–0.96	0.75–0.94
Physical functioning score		
ICC	0.77	0.94
95% CI	0.57–0.88	0.89–0.97
Role functioning score		
ICC	0.81	0.84
95% CI	0.65–0.90	0.69–0.92
Emotional functioning score		
ICC	0.80	0.88
95% CI	0.62–0.90	0.76–0.94
Cognitive functioning score		
ICC	0.87	0.90
95% CI	0.75–0.93	0.79–0.95
Social functioning score		
ICC	0.83	0.73
95% CI	0.68–0.91	0.51–0.86
Fatigue score		
ICC	0.66	0.85
95% CI	0.40–0.82	0.71–0.93
Nausea and vomiting		
ICC	0.52	0.88
95% CI	0.20–0.73	0.76–0.94
Pain		
ICC	0.88	0.91
95% CI	0.76–0.94	0.82–0.95
Dyspnoea		
ICC	0.69	0.83
95% CI	0.45–0.84	0.67–0.91
Insomnia		
ICC	0.75	0.88
95% CI	0.55–0.87	0.76–0.94
Appetite loss		
ICC	0.87	0.84
95% CI	0.74–0.93	0.69–0.92
Constipation		
ICC	0.87	0.90
95% CI	0.75–0.93	0.81–0.95
Diarrhoea		
ICC	0.68	0.90
95% CI	0.44–0.83	0.80–0.95
Financial difficulties		
ICC	0.62	0.74
95% CI	0.34–0.79	0.52–0.87

## Discussion

This study aimed to develop and validate a voice script for phone administration of the QLQ-C30 and evaluate its equivalence to paper administration in a sample of patients actively undergoing cancer treatment. During pilot-testing, the voice script was deemed understandable and relevant with minimal comments received from patients.

Results from the final sample of patients included in the equivalence testing indicated good equivalence between paper and phone administration modes, with all total ICC scores above the 0.70 threshold, ranging from 0.72 to 0.90. In the evaluation of paper administration first, two ICC scores were found to be below the 0.70 threshold, for nausea and vomiting (ICC 0.55; 95% CI 0.24–0.76) and financial difficulties (ICC 0.60; 95% CI 0.31–0.79). When comparing differences in means at the domain score level, the differences were still well below 10 points for the comparison of both administration modes, suggesting minimal clinically meaningful differences despite the ICCs [25]. Failure to reach the 0.70 ICC threshold for nausea and vomiting may also reflect the possibility of more ambiguity surrounding the rating of nausea. While vomiting is a more concrete occurrence, and it is unlikely that a patient’s recollection would change over a 2-day timeframe, nausea may be subject to broader interpretation. Moreover, medications are generally readily available to patients, which help to resolve these symptoms on a day-to-day basis, thus indicating that those symptoms can change within a 2-day period. For financial difficulties, the potential source of discrepancy is less clear. There may have been an issue that was not detectable based on the scope of this study, or it may simply be due to random error or noise.

A more general limitation of using ICC to assess equivalence is that the absolute size of a given ICC is dependent on the variation observed within the sample. As such, minimal variation in nausea and vomiting and financial difficulties scores may have contributed to the lower ICCs. Still, the ICCs for nausea and vomiting and financial difficulties were well above 0.50 for paper administration first, indicating that they remain within the minimally acceptable range, especially since the total ICC scores for both scales were over 0.70. It is worth noting that the nausea and vomiting domain score has performed poorly in a previous test–retest study carried out by Hjermstad et al. [26], so there may be other factors influencing that scale, which were not identified in this study. Such factors could also account for the lower ICC score found for nausea and vomiting, when the paper version was administered first.

Differences in mean scores at the domain score level were uniformly minimal, suggesting that, overall, results from both administration modes were equivalent. The relatively short completion time of 8.6 ± 1.9 min for the voice script suggests that it can be integrated into a study protocol with relative ease and minimal patient burden.

Following guidelines from ISPOR’s PRO Mixed Modes Good Research Practices Task Force, and drawing on methodology used in similar PRO equivalence studies [13, 15], this study had a number of strengths. The randomised cross-over design helped to minimise the potential for bias in either one of the administration modes, and the inclusion criteria ensured that the voice script was evaluated and tested by patients for whom it would be relevant and feasible (i.e. those actively undergoing treatment, with the appropriate language level). The final sample of patients was diverse, and sufficiently well-balanced in terms of demographic and clinical characteristics, helping to ensure representativeness across patients and disease types. Analyses were also strengthened by the fact that there were no missing data in either the paper or phone-administered versions of the questionnaire, making the results more easily interpretable.

The decision to decrease the initial ICC threshold from ≥ 0.90 to ≥ 0.70 following the study amendment to include a second wave of testing, is well-supported by robust evidence in the literature, for which an ICC of ≥ 0.70 has also been used to evaluate equivalence in similar studies [13–15].

Moreover, the total ICCs for both waves were largely similar. Significant differences were only observed for nausea and vomiting, which was lower in the first wave, and constipation, which was lower in the second wave of testing. Although the same recruitment procedures and inclusion criteria were applied, and demographic and clinical characteristics were largely comparable between groups, differences in gender and age distribution were found between the two waves of testing. In light of these differences, sensitivity analyses were carried out across all participants by age and gender. While most scores were similar across groups, differences were found in ICC scores for the nausea and vomiting and diarrhoea domain scores by group, with younger patients scoring lower on these domains compared to older patients. Males also scored lower than females on both the nausea and vomiting and physical functioning scales.

Despite these findings, when examining all other ICCs and comparing them across subgroups, no consistent pattern was identified which would support a potential correlation between lower or higher ICCs and the age or gender of patients. Moreover, the limited sample size makes it difficult to draw robust conclusions at the subgroup level. Factors other than age and gender may be related to the experience of disease and treatment, and may help to account for the differences observed; however, such interpretations are beyond the scope of this study, which is limited to the available demographic and clinical data.

Overall, sensitivity analyses showed that differences observed between the two waves of testing are minimal, thereby further supporting the equivalence of paper-and-pen and phone administration modes.

## Conclusions

Results from this study support the equivalence of paper and phone administration modes of the QLQ-C30. In addition to its initial source language (UK English) development, the QLQ-C30 voice script is now available in multiple other languages, with more translations anticipated in the future. By providing an alternative means of questionnaire completion, the QLQ-C30 voice script helps to ensure that the questionnaire remains accessible in multiple formats across a wide range of patients.

## Supplementary Information

Below is the link to the electronic supplementary material.

Supplementary file1 (DOCX 22 kb)
