# Supplementary material for: Equivalence testing of a newly developed interviewer-led telephone script for the EORTC QLQ-C30
Source: Qual Life Res. 2021 Jul 20;31(3):877–88. doi: 10.1007/s11136-021-02955-6 (PMC8921039; doi:10.1007/s11136-021-02955-6)
Supplement: Supplementary file 1 — Supplementary file1 (DOCX 22 kb) [file 11136_2021_2955_MOESM1_ESM.docx]

**APPENDIX A**

A1. Equivalence testing for single items

| QLQ-C30 items | All patients | |  |
| --- | --- | --- | --- |
|  | Paper first  (n=31) | Phone first  (n=32) | Total  (n=63) |
| Item 1 (Strenuous activity)  Weighted Kappa  95% CI | 0.50  0.21-0.79 | 0.73  0.55-0.91 | 0.65  0.50-0.81 |
| Item 2 (Long walk)  Weighted Kappa  95% CI | 0.64  0.41-0.87 | 0.85  0.71-0.99 | 0.77  0.64-0.89 |
| Item 3 (Short walk)  Weighted Kappa  95% CI | 0.85  0.65-1.00 | 0.83  0.62-1.00 | 0.84  0.69-0.99 |
| Item 4 (Stay in bed or chair)  Weighted Kappa  95% CI | 0.51  0.26-0.77 | 0.73  0.55-0.91 | 0.65  0.50-0.81 |
| Item 5 (Assistance)  Weighted Kappa  95% CI | 0.83  0.53-1.00 | 0.72  0.44-1.00 | 0.77  0.56-0.98 |
| Item 6 (Work or daily activities)  Weighted Kappa  95% CI | 0.64  0.44-0.83 | 0.63  0.43-0.83 | 0.64  0.50-0.78 |
| Item 7 (Hobbies)  Weighted Kappa  95% CI | 0.65  0.47-0.82 | 0.68  0.52-0.84 | 0.67  0.55-0.79 |
| Item 8 (Short of breath)  Weighted Kappa  95% CI | 0.73  0.51-0.96 | 0.65  0.43-0.86 | 0.69  0.53-0.85 |
| Item 9 (Pain)  Weighted Kappa  95% CI | 0.78  0.61-0.96 | 0.82  0.68-0.95 | 0.80  0.70-0.91 |
| Item 10 (Rest)  Weighted Kappa  95% CI | 0.63  0.40-0.86 | 0.55  0.33-0.76 | 0.59  0.43-0.74 |
| Item 11 (Trouble sleeping)  Weighted Kappa  95% CI | 0.77  0.62-0.93 | 0.71  0.55-0.87 | 0.74  0.63-0.85 |
| Item 12 (Weak)  Weighted Kappa  95% CI | 0.60  0.38-0.81 | 0.41  0.19-0.63 | 0.52  0.36-0.68 |
| Item 13 (Lack appetite)  Weighted Kappa  95% CI | 0.85  0.68-1.00 | 0.74  0.58-0.89 | 0.79  0.67-0.91 |
| Item 14 (Nausea)  Weighted Kappa  95% CI | 0.68  0.43-0.92 | 0.87  0.73-1.00 | 0.78  0.64-0.93 |
| Item 15 (Vomiting)  Weighted Kappa  95% CI | 0.64  0.09-1.00 | 0.79  0.33-1.00 | 0.71  0.32-1.00 |
| Item 16 (Constipation)  Weighted Kappa  95% CI | 0.87  0.73-1.00 | 0.79  0.65-0.93 | 0.82  0.73-0.92 |
| Item 17 (Diarrhoea)  Weighted Kappa  95% CI | 0.79  0.53-1.00 | 0.75  0.60-0.90 | 0.77  0.63-0.92 |
| Item 18 (Tired)  Weighted Kappa  95% CI | 0.61  0.37-0.84 | 0.66  0.46-0.85 | 0.63  0.48-0.79 |
| Item 19 (Pain and activity)  Weighted Kappa  95% CI | 0.70  0.51-0.89 | 0.68  0.51-0.85 | 0.69  0.56-0.81 |
| Item 20 (Difficulty concentrating)  Weighted Kappa  95% CI | 0.75  0.58-0.92 | 0.65  0.45-0.86 | 0.70  0.57-0.84 |
| Item 21 (Tense)  Weighted Kappa  95% CI | 0.63  0.42-0.84 | 0.62  0.42-0.81 | 0.63  0.49-0.78 |
| Item 22 (Worry)  Weighted Kappa  95% CI | 0.41  0.17-0.65 | 0.58  0.38-0.79 | 0.52  0.37-0.68 |
| Item 23 (Irritable)  Weighted Kappa  95% CI | 0.63  0.43-0.82 | 0.67  0.51-0.83 | 0.65  0.52-0.77 |
| Item 24 (Depressed)  Weighted Kappa  95% CI | 0.65  0.48-0.82 | 0.90  0.79-1.00 | 0.79  0.69-0.89 |
| Item 25 (Difficulty remembering)  Weighted Kappa  95% CI | 0.73  0.53-0.93 | 0.56  0.39-0.74 | 0.64  0.51-0.78 |
| Item 26 (Family life)  Weighted Kappa  95% CI | 0.56  0.34-0.78 | 0.65  0.44-0.86 | 0.60  0.44-0.76 |
| Item 27 (Social)  Weighted Kappa  95% CI | 0.55  0.34-0.76 | 0.65  0.48-0.82 | 0.60  0.47-0.74 |
| Item 28 (Financial)  Weighted Kappa  95% CI | 0.49  0.25-0.72 | 0.78  0.60-0.96 | 0.63  0.48-0.78 |
| Item 29 (Overall health)  Weighted Kappa  95% CI | 0.73  0.58-0.89 | 0.70  0.54-0.86 | 0.72  0.61-0.83 |
| Item 30 (Overall QoL)  Weighted Kappa  95% CI | 0.69  0.56-0.83 | 0.77  0.61-0.94 | 0.74  0.63-0.85 |

**APPENDIX B**

B1. Mean differences in single item scores between paper- and phone-administered versions.

| QLQ-C30 items | All patients | |
| --- | --- | --- |
|  | Paper first  (N=31) | Phone first  (N=32) |
| Item 1 (Strenuous activity)  Mean (SD)  Min - Max | -0.03 (0.66)  -2.00 - 1.00 | -0.13 (0.66)  -2.00 - 2.00 |
| Item 2 (Long walk)  Mean (SD)  Min - Max | 0.10 (0.60)  -1.00 - 2.00 | 0.09 (0.47)  -1.00 - 2.00 |
| Item 3 (Short walk)  Mean (SD)  Min - Max | 0.06 (0.25)  0.00 - 1.00 | -0.03 (0.40)  -2.00 - 1.00 |
| Item 4 (Stay in bed or chair)  Mean (SD)  Min - Max | 0.19 (0.48)  -1.00 - 1.00 | -0.16 (0.45)  -1.00 - 1.00 |
| Item 5 (Assistance)  Mean (SD)  Min - Max | 0.03 (0.18)  0.00 - 1.00 | 0.06 (0.25)  0.00 - 1.00 |
| Item 6 (Work or daily activities)  Mean (SD)  Min - Max | 0.00 (0.52)  -1.00 - 1.00 | -0.19 (0.59)  -1.00 - 1.00 |
| Item 7 (Hobbies)  Mean (SD)  Min - Max | 0.03 (0.55)  -1.00 - 1.00 | -0.16 (0.57)  -1.00 - 1.00 |
| Item 8 (Short of breath)  Mean (SD)  Min - Max | 0.06 (0.51)  -1.00 - 2.00 | -0.25 (0.44)  -1.00 - 0.00 |
| Item 9 (Pain)  Mean (SD)  Min - Max | 0.10 (0.54)  -1.00 - 2.00 | 0.00 (0.44)  -1.00 - 1.00 |
| Item 10 (Rest)  Mean (SD)  Min - Max | -0.03 (0.60)  -2.00 - 1.00 | -0.31 (0.69)  -2.00 - 1.00 |
| Item 11 (Trouble sleeping) |  |  |
| Mean (SD) | 0.03 (0.48) | -0.22 (0.61) |
| Min - Max | -1.00 - 1.00 | -2.00 - 1.00 |
| Item 12 (Weak) |  |  |
| Mean (SD) | 0.06 (0.73) | -0.09 (0.73) |
| Min - Max | -2.00 - 2.00 | -1.00 - 2.00 |
| Item 13 (Lack appetite) |  |  |
| Mean (SD) | 0.10 (0.30) | 0.00 (0.44) |
| Min - Max | 0.00 - 1.00 | -1.00 - 1.00 |
| Item 14 (Nausea) |  |  |
| Mean (SD) | 0.19 (0.48) | -0.09 (0.30) |
| Min - Max | 0.00 - 2.00 | -1.00 - 0.00 |
| Item 15 (Vomiting) |  |  |
| Mean (SD) | 0.06 (0.36) | -0.03 (0.18) |
| Min - Max | 0.00 - 2.00 | -1.00 - 0.00 |
| Item 16 (Constipation) |  |  |
| Mean (SD) | -0.03 (0.31) | -0.06 (0.44) |
| Min - Max | -1.00 - 1.00 | -1.00 - 1.00 |
| Item 17 (Diarrhoea) |  |  |
| Mean (SD) | -0.03 (0.60) | -0.13 (0.42) |
| Min - Max | -3.00 - 1.00 | -1.00 - 1.00 |
| Item 18 (Tired) |  |  |
| Mean (SD) | 0.03 (0.55) | -0.16 (0.51) |
| Min - Max | -1.00 - 1.00 | -1.00 - 1.00 |
| Item 19 (Pain and activity) |  |  |
| Mean (SD) | -0.03 (0.55) | 0.09 (0.64) |
| Min - Max | -1.00 - 1.00 | -1.00 - 2.00 |
| Item 20 (Difficulty concentrating) |  |  |
| Mean (SD) | -0.16 (0.45) | -0.19 (0.59) |
| Min - Max | -1.00 - 1.00 | -2.00 - 1.00 |
| Item 21 (Tense) |  |  |
| Mean (SD) | -0.06 (0.51) | -0.13 (0.75) |
| Min - Max | -1.00 - 1.00 | -2.00 - 2.00 |
| Item 22 (Worry) |  |  |
| Mean (SD) | 0.03 (0.75) | -0.22 (0.75) |
| Min - Max | -1.00 - 2.00 | -2.00 - 1.00 |
| Item 23 (Irritable) |  |  |
| Mean (SD) | -0.13 (0.56) | -0.16 (0.51) |
| Min - Max | -1.00 - 1.00 | -1.00 - 1.00 |
| Item 24 (Depressed) |  |  |
| Mean (SD) | 0.10 (0.54) | -0.03 (0.31) |
| Min - Max | -1.00 - 1.00 | -1.00 - 1.00 |
| Item 25 (Difficulty remembering) |  |  |
| Mean (SD) | -0.03 (0.55) | -0.03 (0.65) |
| Min - Max | -1.00 - 2.00 | -1.00 - 1.00 |
| Item 26 (Family life) |  |  |
| Mean (SD) | 0.39 (0.76) | 0.00 (0.62) |
| Min - Max | -1.00 - 3.00 | -2.00 - 1.00 |
| Item 27 (Social) |  |  |
| Mean (SD) | 0.19 (0.70) | -0.06 (0.67) |
| Min - Max | -2.00 - 1.00 | -2.00 - 1.00 |
| Item 28 (Financial) |  |  |
| Mean (SD) | 0.32 (0.91) | 0.16 (0.37) |
| Min - Max | -2.00 - 2.00 | 0.00 - 1.00 |
| Item 29 (Overall health) |  |  |
| Mean (SD) | -0.02 (0.64) | -0.03 (0.78) |
| Min - Max | -1.00 - 2.00 | -2.00 - 2.00 |
| Item 30 (Overall QoL) |  |  |
| Mean (SD) | -0.06 (0.73) | -0.03 (0.82) |
| Min - Max | -1.00 - 2.00 | -3.00 - 2.00 |
